# Understanding and Resolving Failures in Human-Robot Interaction: Literature Review and Model Development

**DOI:** 10.3389/fpsyg.2018.00861

**Published:** 2018-06-15

**Authors:** Shanee Honig, Tal Oron-Gilad

**Affiliations:** Mobile Robotics Laboratory and HRI Laboratory, Department of Industrial Engineering and Management, Ben-Gurion University of the Negev, Beer Sheva, Israel

**Keywords:** human-robot interaction, failure, user-centered, information processing, context

## Abstract

While substantial effort has been invested in making robots more reliable, experience demonstrates that robots operating in unstructured environments are often challenged by frequent failures. Despite this, robots have not yet reached a level of design that allows effective management of faulty or unexpected behavior by untrained users. To understand why this may be the case, an in-depth literature review was done to explore when people perceive and resolve robot failures, how robots communicate failure, how failures influence people's perceptions and feelings toward robots, and how these effects can be mitigated. Fifty-two studies were identified relating to communicating failures and their causes, the influence of failures on human-robot interaction (HRI), and mitigating failures. Since little research has been done on these topics within the HRI community, insights from the fields of human computer interaction (HCI), human factors engineering, cognitive engineering and experimental psychology are presented and discussed. Based on the literature, we developed a model of information processing for robotic failures (Robot Failure Human Information Processing, RF-HIP), that guides the discussion of our findings. The model describes the way people perceive, process, and act on failures in human robot interaction. The model includes three main parts: (1) communicating failures, (2) perception and comprehension of failures, and (3) solving failures. Each part contains several stages, all influenced by contextual considerations and mitigation strategies. Several gaps in the literature have become evident as a result of this evaluation. More focus has been given to technical failures than interaction failures. Few studies focused on human errors, on communicating failures, or the cognitive, psychological, and social determinants that impact the design of mitigation strategies. By providing the stages of human information processing, RF-HIP can be used as a tool to promote the development of user-centered failure-handling strategies for HRIs.

## Introduction

While substantial effort has been invested in making robots more reliable, experience demonstrates that robots are often challenged by frequent failures. The Mean Time Between Failure (MTBF) for robots in field environments is often within a few hours (Tsarouhas and Fourlas, 2016). Despite this, mobile robots have not yet reached a level of design that allow effective management of faulty or unexpected behavior. In fact, research suggests that the relationship between symptoms and cause of failure is often not clear even to trained roboticists (Steinbauer, 2013). Having to rely on a professional to understand and resolve a robot's faulty behavior is a barrier to acceptance amongst untrained users. Customer support also becomes costly when users are unable to differentiate between technical errors (software bugs or hardware failures) and problems resulting from improper use (misuse; Parasuraman and Riley, 1997) or unrealistic expectations. Moreover, how a robot manages failure influences willingness to use the robot again (Lee et al., 2010), the degree of deterioration in task performance (Ragni et al., 2016), user trust in the robot (Hamacher et al., 2016), and people's perceptions of the robot (Gompei and Umemuro, 2015), suggesting that failure handling may have substantial commercial and economic benefits. Yet, little is known about how to create failure management tools for robots that are appropriate for untrained users. We shed light on this topic, with the goal of developing design tools and design guidelines that facilitate development of robot interactions that enable untrained users to quickly and easily identify and act on failures, while maintaining a positive user experience.

To tackle the challenging problem of failure handling for untrained users, it is first necessary to review the cognitive considerations that critically influence naive users' ability to detect and solve robot failures, and evaluate whether these considerations have been properly addressed in the existing Human-Robot Interaction (HRI) literature. This paper presents a detailed look at the literature in HRI regarding when people perceive and resolve robot failures, how robots communicate failure, how failures influence people's perceptions and feelings toward robots, and how these effects can be mitigated. Since little research has been done on these topics within the HRI community, insights from the fields of Human Computer Interaction (HCI), human factors engineering, cognitive engineering and experimental psychology are presented and discussed. To the best of our knowledge, a thorough review of robotic failure handling from a user-centered perspective has not yet been conducted. Based on the literature, we developed a model of information processing for robotic failures (the Robot Failure Human Information Processing Model, RF-HIP) that guides the discussion of our findings. As robots become more present in day-to-day life, especially for elderly users who are inexperienced with robotic applications (Beer and Takayama, 2011), we anticipate that such reviews and models will become increasingly useful. Researchers could use them to better understand what influences failure handling in HRIs, to identify possible knowledge gaps and to promote future research directions. Roboticists, engineers, and designers could use them to guide design choices that will increase user acceptance and decrease customer support costs. Policy makers could use them to decide on standards for the necessary failure-handling techniques required to make robots safe for general use.

The paper is organized as follows: first, the types of failures that may occur during HRIs are discussed. Second, search criteria and an overview of the relevant HRI literature that matched these criteria is presented. Third, cognitive determinants that are likely to influence a person's ability to perceive and resolve failures are combined with current research in robotic user-centered failure handling to create a model of information processing. Finally, gaps in the HRI literature are presented and discussed.

## Defining and classifying errors

Various definitions exist for the terms “failure,” “error,” and “fault.” In line with (Laprie, 1995; Carlson and Murphy, 2005; Steinbauer, 2013; Brooks, 2017), we adopted terminology in which *failure* refers to “a degraded state of ability which causes the behavior or service being performed by the system to deviate from the ideal, normal, or correct functionality” (Brooks, 2017). This definition includes both perceived failures, unexpected behavior and actual failures, which is consistent with findings that suggest that intentional yet unexpected or incoherent behaviors are sometimes interpreted as erroneous (Short et al., 2010; Lemaignan et al., 2015). Failures result from one or more *errors*, which refer to system states (electrical, logical, or mechanical) that can lead to a failure. Errors result from one or more *faults*, which refer to anything that causes the system to enter an error state. For example, a robot may experience a *failure* resulting from an *error* in face-recognition, caused by poor illumination (*fault*).

It is improbable to identify all possible types of robotic failures since mobile robots operate in unstructured changing environments with a wide variety of possible interactions. Yet, several taxonomies for classifying errors and failures have been proposed. Laprie (1995) classified failures according to severity, defining *benign failures* (failures whose consequences are comparable to the benefits of the service they are preventing) and *catastrophic failures* (failures with a higher cost by one or more orders of magnitude than the service). Ross (Ross et al., 2004) categorized system errors according to failure recoverability, defining *anticipated errors* (when the agent backtracks through the plan to achieve the same goal through an alternate course of action), *exceptional errors* (when the current plan cannot cope with the failure, and re-planning can be done to formulate a strategy to achieve the original goal)*, unrecoverable errors* (when the current plan cannot cope with the error and re-planning cannot be done), and *socially recoverable errors* (when the agent can continue on with the original plan with appropriate assistance from other agents within its environment). Giuliani et al. (2015) classified failures according to their type, defining *technical failures* (caused by technical shortcomings of the robot) and *social norm violations* (when the robot deviates from the social script or uses inappropriate social signals, e.g., looking away from a person while talking to them).

Carlson and Murphy (2005) devised an extensive error classification taxonomy by analyzing how Unmanned Ground Vehicles (UGVs) failed in the field using studies from urban search and rescue and military field applications. The classification, based on Laprie (1995) and Norman (2002) categorized errors according to the source of failure (the fault), and included two main categories: (1) *physical failures*, which are failures caused by physical errors in the system's effectors, sensors, control system, power sources, or communications, and (2) *human failures*, which are caused by human-made errors. They further classified physical failures according to *severity* (*terminal failure*—terminates the system's current mission; *nonterminal failures*—degrades its ability to perform its mission) and *repairability* (*field repairable—*repairable with tools that accompany the system in the field; *nonfield repairable*—cannot be repaired with tools that accompany the system in the field), and human failures according to *design failures* (errors introduced during design, construction, or post-production modifications, e.g., programmed to greet people with “goodbye”) and *interaction failures* (errors introduced by unintended violations of operating procedures). Interaction failures included *mistakes* (performing an action that is wrong), and *slips* [attempting to do the right thing unsuccessfully, e.g., accidentally pressing the wrong button (Barakova et al., 2015)].

While the (Carlson and Murphy, 2005) taxonomy is extensive, there are additional interaction failures that were not accounted for. For example, it did not consider other types of human errors, such as *lapses*, which occur as a result of lapses of memory and/or attention (e.g., forgetting to turn the robot off), and *deliberate violations*, which are intentional illegitimate actions (e.g., directing the robot to run into a wall) (Reason, 1990). Three main taxonomies of human errors are frequently cited in the literature (Stanton and Salmon, 2009): (1) Norman's error categorization (Norman, 1981), which divides human errors into those that result from misinterpretations of the situation, those that result from faulty activation of schemas (knowledge structures) due to similar trigger conditions, and those that result from activating schemas too early, too late, or not at all; (2) Rasmussen's error categorization (Rasmussen, 1982), which divides human errors by the level of cognitive control within which they occur (skill-, rule-, or knowledge-based), and (3) Reason's categorization (Reason, 1990), which builds on Rassmussen's ideas and divides human errors into slips, lapses, mistakes and violations (described above). Moreover, the (Carlson and Murphy, 2005) taxonomy doesn't consider uncertainties in the interaction that result from varying environments and other agents. (Sutcliffe and Rugg, 1998) described 10 environmental and social factors that may increase the likelihood of errors, and classified them into *group level judgement, working environment*, and *organizational flaws*.

Steinbauer (2013) collected information regarding failures that occurred to teams in RoboCup competitions, and classified them into four categories: *Interaction* (problems that arise from uncertainties in the interaction with the environment, other agents, and humans), *algorithms* (problems in methods and algorithms), *software* (design and implementation faults of software systems), and *hardware* (physical faults of the robotic equipment). They used several attributes to classify faults and their properties, including the fault's relevance to different robotic systems (*relevance*), the context in which the fault occurred (*condition*), indicators used to identify the failure (*symptoms*), how the failure impacted the mission (*impact*: non-critical, repairable, and terminal), and the frequency of the occurrence of a fault (*frequency*: never, sporadic, regularly, frequently).

Brooks (2017), based on Lutz and Woodhouse (1999), identified two main types of failure: communication failures and processing failures. Communication failures are related to data being passed between modules, including *missing data* (incomplete messages or dropped packets), *incorrect data* (data generated incorrectly or distorted during transmission), *bad timing* (data sent too early, before the receiver is ready to handle it, or too late, causing delays in reaction), and *extra data* (data sent multiple times but only expected once, or sending larger messages than expected). Processing failures include *abnormal terminations*, that could happen due to unhandled exceptions, segmentation fault, or dead-lock; *missing events*, that could happen when a conditional statement is not triggered or a callback or interrupt never fires; *incorrect logic* due to bad assumptions or unforeseen conditions; and *timing or ordering*, where events take place in a different order than expected or a waiting period times-out before information arrives.

**Figure 1:**
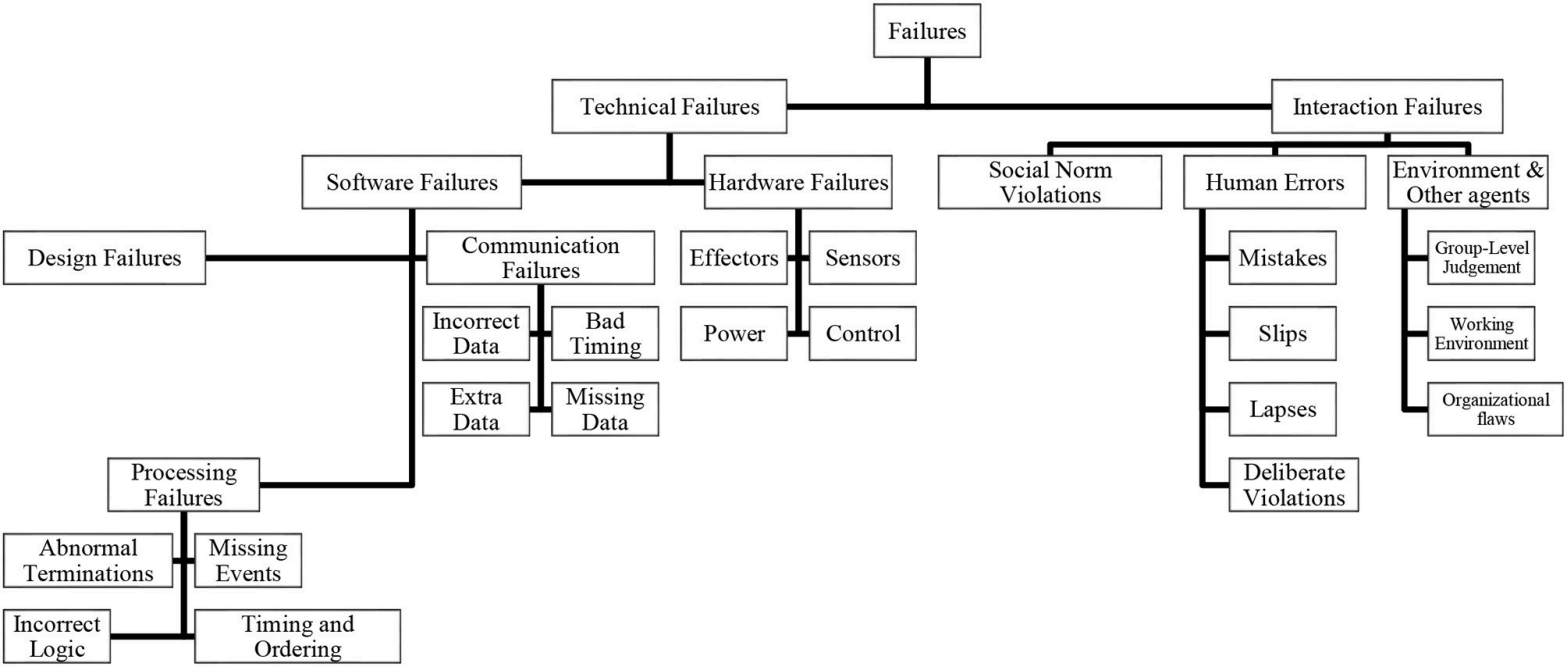
A human-robot failure taxonomy.

We propose an inclusive human-robot failure taxonomy that combines the above system and human oriented classifications (Figure 1). According to this taxonomy, the main distinction is between two types of failures: *technical failures* and *interaction failures*. *Technical failures* are caused either by hardware errors or problems in the robot's software system. Software errors are further classified into design failures, communication failures, and processing failures. Following Steinbauer's categorization (Steinbauer, 2013), *interaction failures* refer to problems that arise from uncertainties in the interaction with the environment, other agents, and humans. These include social norm violations and various types of human errors as noted in Reason (1990). Each failure event, regardless of its source, can be categorized by the following attributes:

- ***Functional Severity:*** criticality of the failure to the robot's functioning (non-critical, recoverable, terminal).
- ***Social Severity:*** criticality of the failure to future acceptance of the robot's services (non-critical, recoverable, unrecoverable).
- ***Relevance:*** relevance of the fault to different robot systems, which can be high (relevant to almost all robotic systems), medium (relevant only to some robotic systems), or low (highly specialized failures).
- ***Frequency:*** how often the failure occurs (never, sporadic, regularly, frequently).
- ***Condition:*** the context in which the fault and failure occurred.
- ***Symptoms***: indicators used to identify the failure.

## Literature review on user-centered failure handling

Various search engines were used to conduct the online literature search on human-centered failure handling in robots, including Google Scholar, IEEE, ACM, Science Direct, Springer, Sage Journals, Taylor & Francis Online, and Cambridge Core. Robotics conferences and journals covered in this search include ICRA, IROS, RO-MAN, SMC, Robotics and Autonomous Systems, Human Machine Systems, HRI, International Journal of Social Robotics, Autonomous Robots, International Journal of Robotics Research, Robotica, Intelligent Robots and Systems, and Advanced Robotics, amongst others. Keywords used were: robot, error, failure, recovery, reliability. Included in the review are articles that address robotic failure-handling from the perspective of the human operator, user or bystander, rather than from a systems perspective. That is, we focused on studies that evaluated some aspect of the bilateral relationship between end-user's needs, wants and limitations and robotic failure. Articles that dealt with errors without addressing the user or the interaction were not included in the review. Given the vast amount of research on technical considerations of robot reliability and error handling, we cannot claim our search to be exhaustive, however given the large number of resources surveyed, we do believe it is indicative of current trends.

**Figure 2:**
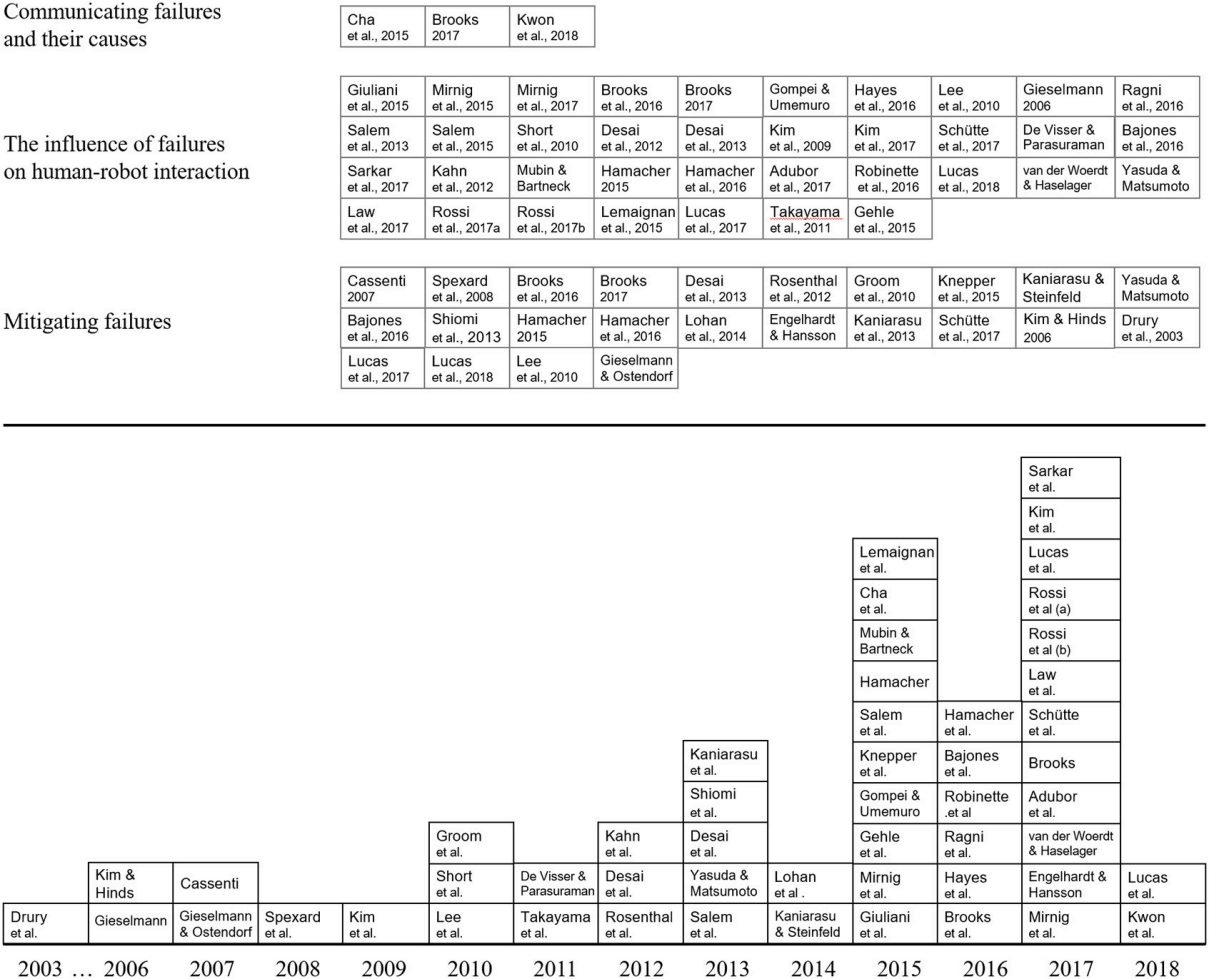
Distribution of user-centered failure handling by topic (**Top**) and by publication year (**Bottom**).

Figure 2 shows the result of the literature search of HRI articles that evaluated some aspect of user-centered failure handling. Altogether, 52 relevant papers were identified, where 40 of them were published in conference proceedings, 8 in academic journals, 1 doctoral dissertation, 2 theses, and 1 technical report. Papers were classified into three main topics: (a) communicating failures and their causes, i.e., how should a robot communicate to its user and bystanders that an error has occurred; (b) the influence of failures on HRI, i.e., how do failures influence user perceptions of the robot and user behavior; and (c) mitigating failures, i.e., approaches on how to mitigate the negative effects of failure on HRIs. The following sections provide an overview of methodologies used in the literature, including the types of errors and symptoms studied, evaluation methods and metrics, the types of robotic systems used, and experimental environments.

### Errors and symptoms studied

Almost all errors researched in the literature exemplified technical failures (e.g., Gieselmann, 2006; Kim and Hinds, 2006; Gieselmann and Ostendorf, 2007; Spexard et al., 2008; Kim et al., 2009; Groom et al., 2010; Lee et al., 2010; Takayama et al., 2011; Desai et al., 2012, 2013; Kahn et al., 2012; Rosenthal et al., 2012; Shiomi et al., 2013; Yasuda and Matsumoto, 2013; Kaniarasu and Steinfeld, 2014; Lohan et al., 2014; Cha et al., 2015; Gehle et al., 2015; Giuliani et al., 2015; Gompei and Umemuro, 2015; Hamacher, 2015; Knepper et al., 2015; Mirnig et al., 2015, 2017; Mubin and Bartneck, 2015; Salem et al., 2015; Bajones et al., 2016; Brooks et al., 2016; Hamacher et al., 2016; Hayes et al., 2016; Ragni et al., 2016; Robinette et al., 2016; Engelhardt and Hansson, 2017; Law et al., 2017; Sarkar et al., 2017; van der Woerdt and Haselager, 2017; Kwon et al., 2018). Only a few evaluated the impact of social norm violations (e.g., Short et al., 2010; Salem et al., 2013; Giuliani et al., 2015; Mirnig et al., 2015, 2017; van der Woerdt and Haselager, 2017), and none focused on human errors. Some articles did not specify the type of error used (e.g., Ross et al., 2004; Cassenti, 2007).

A robot's failure symptoms in the literature include the robot not completing a given task (e.g., Takayama et al., 2011; Rosenthal et al., 2012; Brooks et al., 2016; Robinette et al., 2016; Mirnig et al., 2017; Kwon et al., 2018), running into obstacles (e.g., Brooks et al., 2016), performing the wrong action (e.g., Kim et al., 2009; Lee et al., 2010; Desai et al., 2012, 2013; Yasuda and Matsumoto, 2013; Kaniarasu and Steinfeld, 2014; Gehle et al., 2015; Mubin and Bartneck, 2015; Salem et al., 2015; Brooks et al., 2016; Hayes et al., 2016; Robinette et al., 2016; Mirnig et al., 2017; Sarkar et al., 2017; van der Woerdt and Haselager, 2017), performing the right action incorrectly or incompletely (e.g., Takayama et al., 2011; Shiomi et al., 2013; Cha et al., 2015; Hamacher, 2015; Brooks et al., 2016; Hamacher et al., 2016; Adubor et al., 2017; Sarkar et al., 2017; van der Woerdt and Haselager, 2017; Kwon et al., 2018), producing no action or speech (irresponsiveness) (e.g., Gieselmann, 2006; Lohan et al., 2014; Bajones et al., 2016; Robinette et al., 2016; Lucas et al., 2017, 2018), timing speech improperly (e.g., Mirnig et al., 2017), failing to produce speech (e.g., Gieselmann and Ostendorf, 2007; Mirnig et al., 2017), producing inappropriate speech or erroneous instruction (e.g., Gieselmann, 2006; Gieselmann and Ostendorf, 2007; Short et al., 2010; Gehle et al., 2015; Gompei and Umemuro, 2015; Lucas et al., 2017, 2018; Mirnig et al., 2017; Sarkar et al., 2017), repeating statements or body movements (e.g., Gieselmann and Ostendorf, 2007; Spexard et al., 2008; Lucas et al., 2017; Kwon et al., 2018), producing unexpected or erratic behavior (e.g., Kim and Hinds, 2006; Spexard et al., 2008; Short et al., 2010; Desai et al., 2012; Salem et al., 2013, 2015; Lemaignan et al., 2015; Robinette et al., 2016; van der Woerdt and Haselager, 2017), making knowledge-based mistakes (e.g., Groom et al., 2010; Short et al., 2010; Kahn et al., 2012; Rosenthal et al., 2012; Salem et al., 2015; Hayes et al., 2016; Ragni et al., 2016; Engelhardt and Hansson, 2017; Law et al., 2017), overtly stating there is a problem (e.g., Spexard et al., 2008; Bajones et al., 2016; Lucas et al., 2018), asking for help (e.g., Ross et al., 2004; Hüttenrauch and Severinson-Eklundh, 2006; Spexard et al., 2008; Rosenthal et al., 2012; Yasuda and Matsumoto, 2013; Knepper et al., 2015; Bajones et al., 2016; Srinivasan and Takayama, 2016), producing body language associated with failure (e.g., Takayama et al., 2011), and questioning for additional information (e.g., Gieselmann, 2006; Lucas et al., 2018).

### Evaluation methods and metrics

Error recovery strategies and reactions to errors have been evaluated using surveys (e.g., Lee et al., 2010; Takayama et al., 2011; Cha et al., 2015; Brooks et al., 2016; Adubor et al., 2017; Kim et al., 2017; Rossi et al., 2017b; van der Woerdt and Haselager, 2017; Kwon et al., 2018), video analysis of HRIs (e.g., Giuliani et al., 2015; Mirnig et al., 2015), and unstructured observational studies (e.g., Gieselmann, 2006; Gehle et al., 2015), however most studies used controlled user experiments (e.g., Spexard et al., 2008; Short et al., 2010; Desai et al., 2013; Salem et al., 2013, 2015; Gompei and Umemuro, 2015; Knepper et al., 2015; Hayes et al., 2016; Ragni et al., 2016; Robinette et al., 2016; Mirnig et al., 2017; Lucas et al., 2018). One study introduced an idea on how to improve situation awareness (SA; see Comprehension and Memory section) in erroneous situations without any formal evaluation (Cassenti, 2007).

User perceptions of the robot that have been evaluated in erroneous situations include the robot's perceived agency (Lemaignan et al., 2015; van der Woerdt and Haselager, 2017), predictability (van der Woerdt and Haselager, 2017), apologeticness (Shiomi et al., 2013), moral accountability (Kahn et al., 2012), friendliness (Groom et al., 2010; Shiomi et al., 2013; Kim et al., 2017), propensity to damage (van der Woerdt and Haselager, 2017), trustworthiness (Gompei and Umemuro, 2015; Brooks et al., 2016; Hamacher et al., 2016; Rossi et al., 2017a; Sarkar et al., 2017; van der Woerdt and Haselager, 2017; Kwon et al., 2018), likeability (Groom et al., 2010; Salem et al., 2013; Bajones et al., 2016; Engelhardt and Hansson, 2017; Mirnig et al., 2017; Sarkar et al., 2017), reliability (Short et al., 2010; Salem et al., 2015), familiarity (Gompei and Umemuro, 2015), anthropomorphism (Lee et al., 2010; Salem et al., 2013, 2015; Lemaignan et al., 2015; Mubin and Bartneck, 2015; Mirnig et al., 2017; Sarkar et al., 2017), animacy (Engelhardt and Hansson, 2017; Sarkar et al., 2017), technical competence (Groom et al., 2010; Short et al., 2010; Desai et al., 2013; Salem et al., 2015; Brooks et al., 2016; Engelhardt and Hansson, 2017; Sarkar et al., 2017), dependability (Brooks et al., 2016), intelligence (Mubin and Bartneck, 2015; Salem et al., 2015; Bajones et al., 2016; Engelhardt and Hansson, 2017; Mirnig et al., 2017; Sarkar et al., 2017), belligerence (Groom et al., 2010) and safety (Salem et al., 2015; Adubor et al., 2017; Sarkar et al., 2017). Studies have also evaluated the effects of errors on engagement (Lemaignan et al., 2015; Law et al., 2017), future contact intensions with the robot (Short et al., 2010; Salem et al., 2013, 2015; Brooks et al., 2016; Robinette et al., 2016; Kwon et al., 2018), the robot being a good teammate (Kwon et al., 2018), psychological closeness with the robot (Salem et al., 2015; Sarkar et al., 2017), rapport and persuasion (Lucas et al., 2018), creating a shared reality (Salem et al., 2013), compliance (Rosenthal et al., 2012; Salem et al., 2015; Robinette et al., 2016; Mirnig et al., 2017), attitudes toward robots (Salem et al., 2013; Gompei and Umemuro, 2015; Kim et al., 2017; Sarkar et al., 2017), and participant's emotional state (e.g., comfortable, safe, relaxed, confused) (Groom et al., 2010; Yasuda and Matsumoto, 2013; Hamacher, 2015; Robinette et al., 2016).

The quality of error recovery and communication strategies have been evaluated using various performance metrics, including whether users managed to resolve the problems (Spexard et al., 2008), attribution of blame (Kim and Hinds, 2006), the frequency of use of recovery feature (Spexard et al., 2008), the number of error-free user interactions (Gieselmann and Ostendorf, 2007; Knepper et al., 2015), time per repair (Rosenthal et al., 2012; Knepper et al., 2015; van der Woerdt and Haselager, 2017), time until task completion (De Visser and Parasuraman, 2011; Rosenthal et al., 2012; Schütte et al., 2017), user comfort (Engelhardt and Hansson, 2017), user satisfaction (Gieselmann and Ostendorf, 2007; Shiomi et al., 2013), task performance and completion (Gieselmann and Ostendorf, 2007; De Visser and Parasuraman, 2011; Desai et al., 2013; Salem et al., 2013; Knepper et al., 2015; Brooks, 2017; Schütte et al., 2017), workload (Brooks, 2017), confidence (De Visser and Parasuraman, 2011; Brooks, 2017), comprehension of information (Brooks, 2017; Kwon et al., 2018), the number of times participant had to stop their primary task to handle the robot (Brooks, 2017), trust in robot (De Visser and Parasuraman, 2011; Rosenthal et al., 2012; Hamacher et al., 2016), the participant's emotional state (Groom et al., 2010) and their influence on user impressions of the robot (Groom et al., 2010; Shiomi et al., 2013; Bajones et al., 2016; Engelhardt and Hansson, 2017; Kwon et al., 2018). Brooks (2017) devised a measurement scale of people's reaction to failure called the REACTION scale, which claims to compare different failure situations based on the severity of the failures, the context risk involved, and effectiveness of recovery strategy. Rossi et al. (2017b) found that people, regardless of age or gender, are fairly consistent in how they rate the severity of robot errors.

The method of measuring each criterion varied; to assess the quality of interaction, research teams mainly used custom made questionnaires with Likert scales and unstructured interviews with a large variety of different questions (e.g., Kim and Hinds, 2006; Short et al., 2010; Rosenthal et al., 2012; Desai et al., 2013; Knepper et al., 2015; Hayes et al., 2016; Robinette et al., 2016; Kwon et al., 2018; Lucas et al., 2018). The most common structured and validated questionnaires used include the Godspeed questionnaire (used in Salem et al., 2015; Bajones et al., 2016; Engelhardt and Hansson, 2017; Mirnig et al., 2017; Sarkar et al., 2017) and NASA TLX (used in Desai et al., 2012, 2013; Hamacher, 2015; Hamacher et al., 2016; Brooks, 2017). Some evaluations were done using video-analysis (Kahn et al., 2012; Hamacher et al., 2016; Sarkar et al., 2017); looking at behavioral data (Kahn et al., 2012; Bajones et al., 2016; Hamacher et al., 2016; Sarkar et al., 2017), verbal statements made during the experiment (Kahn et al., 2012; Bajones et al., 2016; Hamacher et al., 2016), and the number and type of errors made (Bajones et al., 2016). About half of the experimental studies were performed using the Wizard-of-Oz technique (Riek, 2012) (e.g., Gieselmann, 2006; Groom et al., 2010; Short et al., 2010; Kahn et al., 2012; Rosenthal et al., 2012; Yasuda and Matsumoto, 2013; Mubin and Bartneck, 2015; Lucas et al., 2018), and half programmed erroneous behavior to be performed automatically (e.g., Gehle et al., 2015; Gompei and Umemuro, 2015; Hamacher, 2015; Hayes et al., 2016). Only a few studied unplanned failures (e.g., Giuliani et al., 2015; Knepper et al., 2015; Mirnig et al., 2015).

The number of participants used in each study varied, however with the exception of Gieselmann (2006), all had more than 10, which is arguably sufficient to obtain meaningful results through user studies (Nielson, 2000). Most experiments were done on Americans (21) and Europeans (18). Few studies involved non-Western participants (Shiomi et al., 2013; Yasuda and Matsumoto, 2013; Gompei and Umemuro, 2015; Kim et al., 2017), and only one evaluated cross-cultural differences (Rossi et al., 2017a). Participants varied in age, however most studies were primarily implemented on younger adults. One study evaluated children (Lemaignan et al., 2015); none focused on elderly participants above the age of 75. With the exception of seven studies, the distribution between male and female participants was relatively equal (more equal than 60–40%). Sixteen (31%) of the studies evaluated participants with little experience with robots, 2 (3.8%) studies evaluated experienced participants, and 30 (58%) studies did not state the participants' level of experience with robots. Only four studies (7.7%) evaluated both experienced and inexperienced participants (Hamacher, 2015; Hamacher et al., 2016; Rossi et al., 2017a; Lucas et al., 2018).

### Robotic systems

A wide variety of robotic systems are used to study human centered failure handling. NAO was by far the most commonly used robot (Gehle et al., 2015; Giuliani et al., 2015; Gompei and Umemuro, 2015; Mirnig et al., 2015, 2017; Engelhardt and Hansson, 2017; van der Woerdt and Haselager, 2017; Lucas et al., 2018), however several other off-the-shelf solutions were used, including BIRON (Spexard et al., 2008), Kuka youBots (Knepper et al., 2015), iRobot ATRV-JR (Desai et al., 2012), Robovie-mR2 (Shiomi et al., 2013), Snackbot (Lee et al., 2010), and Baxter (Adubor et al., 2017; Sarkar et al., 2017). Several systems were custom made for the purpose of the research (Yasuda and Matsumoto, 2013; Lohan et al., 2014; Lemaignan et al., 2015; Mubin and Bartneck, 2015). About half of the 52 studies used humanoid robots [robots that possess some human-like features (Walters et al., 2008)], and half used mechanoid robots [robots that are machine-like in appearance (Walters et al., 2008)].

### Environment

Experimental evaluations were mostly done indoors, with single-persons (86%). Only one study evaluated robotic failures in outdoor environments (Giuliani et al., 2015), and five of the studies evaluated robotic failures indoors when more than one person was present (Kim and Hinds, 2006; Rosenthal et al., 2012; Gehle et al., 2015; Lemaignan et al., 2015; Bajones et al., 2016). With the exception of Cassenti (2007), which proposed a strategy for helping users recover from errors after prolonged time in which no interaction with the robot was made, all of the studies focused on errors that occurred during interaction with the robot.

## A unified information processing model for user centered failure handling

In order to develop interactions that enable untrained users to easily identify and solve failures, it is critical to consider cognitive factors that influence the ability to perceive and act upon a robotic failure. Interacting with a robot in a moment of failure is inherently an information-processing task—the user must perceive information from the robot and environment, process it to identify if an error has occurred, recall what can be done to fix it or enter a command to obtain additional information, select and then execute responses based on that information. Thus, for failure-handling management tools to be easy to use, the human-robot interface must be designed to meet the information processing capabilities of users.

There are many theories regarding how people process information (e.g., McClelland, 1979; Card et al., 1983, 1986; Miller, 1988; Kieras and Meyer, 1997). One information-processing model that seems particular relevant is the Communication-Human Information Processing (C-HIP) Model (Wogalter, 2006a), which describes the way people process warnings. In situations of failure, indicators from the robot, user and environment can be viewed as warnings of the robot's degraded state of ability. The model includes three main parts: (1) sending the warning, (2) processing it by the receiver, and (3) acting. The parts are described using nine stages that must be completed for people to be compliant with a warning. A bottleneck at any given stage can impede on processing at subsequent stages, and feedback from later stages and additional sources (such as environmental and personal attributes of the receiver) can affect processing in earlier stages.

**Figure 3:**
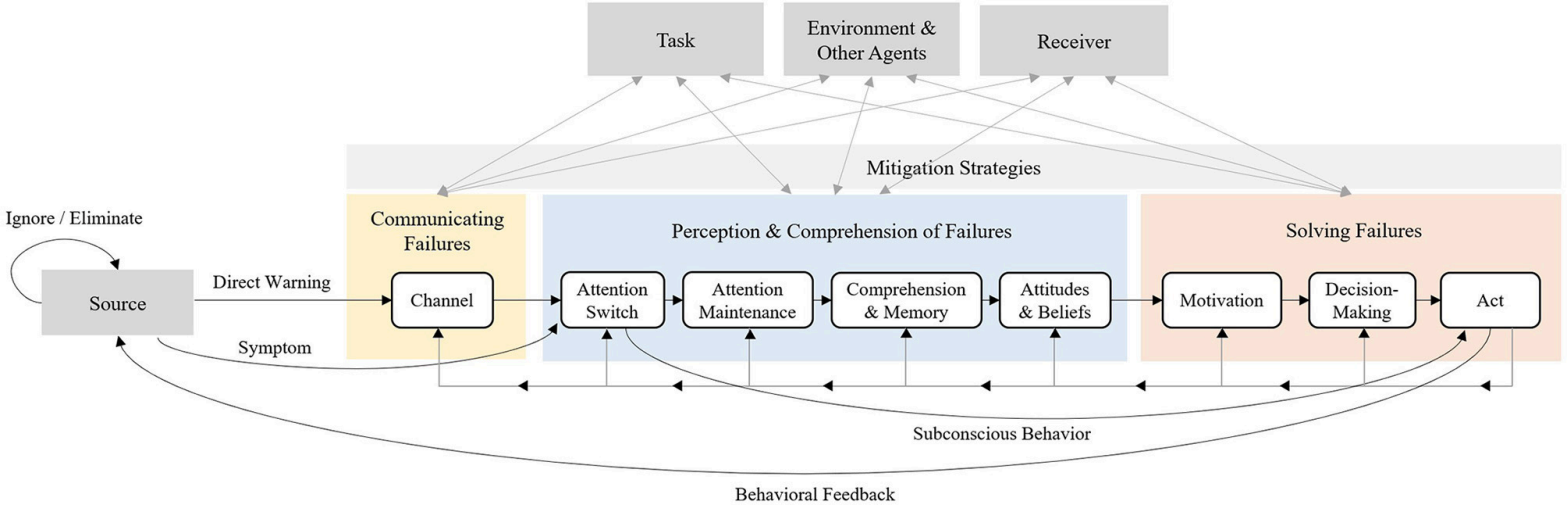
The RF-HIP Model.

After reviewing the cognitive considerations that influence people's ability to detect and solve robot failures, as well as the current literature in failure handling in HRIs, we developed an information processing model called the Robot Failure Human Information Processing (RF-HIP) Model, modeled after C-HIP (Wogalter, 2006a), to describe the way people perceive, process, and act on failures in human robot interactions (Figure 3). By providing the stages of information processing and factors that influence them, RF-HIP can be used as a tool to systematize the assessment process involved in determining why a particular approach of handling failure is successful or unsuccessful in order to facilitate better design. The model, which will be used to guide the presentation of the relevant literature, includes three main parts: (1) communicating failures, (2) perception and comprehension of failures, and (3) solving failures. Each part contains several stages, all heavily influenced by contextual considerations (the source, task, receiver, environment and other agents) and mitigation strategies. The model differs from C-HIP in three primary ways: (1) there is a separate stage for decision making, (2) it accounts for unplanned failure indicators (symptoms) and for subconscious behavior, and (3) it highlights the bilateral relationship between all stages of information processing, contextual factors and mitigation strategies. The components of the model are discussed in the following sections.

### Source

The source is the transmitter of symptoms indicative of a failure. The source of failure is typically the robot, however it could also be the user or other humans in the environment (e.g., in case of human error or when a person produces behavioral responses to robot failure). In situations where a symptom is identified by the source, the source must determine whether it can handle it on its own by ignoring or eliminating the problem, or whether it needs to produce a warning of the symptom to others. If the failure is technical, there are several automatic methods that can be used to detect the error (e.g., Murphy and Hershberger, 1999; Canham et al., 2003) and automatically determine the appropriate recovery method, without involving human assistance (e.g., Murphy and Hershberger, 1999; Mendoza et al., 2015). Several methods also exist to predict and resolve human error in HCI that could be applied to robots (e.g., Embrey, 1986; Baber and Stanton, 1994). Sometimes the symptom is itself a type of warning that is outwardly projected (e.g., the robot's wheel falling off), so the receiver perceives it without the source actively deciding on how to communicate the failure. In such cases, the source may not always be aware of the symptom (e.g., a robot may not be aware when it deviates from social norms).

Warnings can be direct or indirect: a direct warning occurs when the person is directly exposed to the symptom or to a warning from the source, whereas an indirect warning is received in other ways (e.g., learning about the problem from a family member). Various characteristics of the source influence perceived beliefs, credibility, and relevance of symptoms and warnings (Wogalter, 2006a).

### Communicating failures

#### Channel

The channel is the medium and modality which the source uses to transmit information regarding a failure to receivers. While some robot failures can be detected through changes in the robot's behavior or posture (e.g., Takayama et al., 2011; Kwon et al., 2018), changes in the robot's physicality (e.g., a wheel falling off), or changes in the user's behavior (see section Act), other issues (e.g., missing data) produce no obvious symptoms. Moreover, overt changes in robotic behavior may remain undetected by users as a result of poor situation awareness, inexperience with the robot, or lack of supervision (Brooks, 2017). Consequently, various methods have been suggested to intentionally communicate failures and their causes to users and bystanders of robotic systems when possible. If the source identifies a need for a direct warning, it must determine how the relevant agents should be warned. Depending on the source, different channels of communication and delivery methods will be possible.

##### Visual indicators on robot

Brooks (2017) investigated the use of standardized icons displayed on the body of a robot as a method of conveying information about an autonomous robot's internal system state. Specifically, they attempted to convey information about whether the robot is safe to be around and whether it is working properly using five target messages (ok, help, off, safe, and dangerous). Results indicated that icons are a viable method for communicating system state information to untrained bystanders.

Other types of on-robot visual indicators have also been used to indicate robotic errors. One approach is using light (or lack of it)—the Neato robotic vacuum cleaners display an amber light around the main button when it cannot start cleaning^1^; Baraka et al. (2016) used flashing red lights to indicate path obstructions; and Robinette et al. (2016) turned off the robot's lights to indicate inoperability. Another common method is using on-robot screen displays. In Sarkar et al. (2017), Baxter's screen showed a sad smiley face with explanatory text whenever an error was made. Similarly, Jibo^2^ (a personal assistant robot) shows an error code and message on its screen whenever there is an issue^3^.

The primary advantage of using visual indicators on the robot to display failure states is that their placement allows the message to be communicated not only to the robot operator but also to bystanders without any mediating artifacts. Another advantage is that insights and design principles from human factors and HCI literature (e.g., Nielsen, 2001; Wogalter and, 2006c; Egelman et al., 2009; Bauer et al., 2013) could be used as inspiration. There are, however, disadvantages to using visual indicators on the robot. For one, visual indicators on the robot can only influence people who are actively looking and paying attention to the robot. Remote operators and people performing multiple tasks may not notice the indicators in time to act upon them, which is particularly important in failure situations. Second, the message could at times be occluded, depending on the robot's speed and posture relative to the human observer. Third, icons and status lights can effectively convey only simple messaging that represent distinct alternative states of the robot. Screens on the robot can communicate more complex information, however it requires the user to physically come close to the robot, which may not always be safe for certain types of failure. Lastly, the public nature of such indicators may not always be socially appropriate—people may feel uncomfortable having others know about certain errors taking place. For example, a robot unable to track the users' legs because they are too wide or narrow relative to its expectations may cause embarrassment.

##### Secondary screens

Another method of communicating a robot's failure state is by using a secondary screen (such as a smartphone) to provide additional information about the robot. This strategy is one of the most popular in today's commercial robots (e.g., Kuri^4^, iRobot Home Robots^5^, Neato Robotics^6^) and has several advantages: (1) it enables users to interact with the robot using familiar methods of interaction, (2) complex information can be more easily conveyed on-screen, and (3) status information can be accessed remotely and covertly. The main disadvantage of this method is that it inherently shifts the user's eyes and attention away from the robot and from the tasks they are performing, which hinders situation-awareness and could be dangerous in threatening situations. Cassenti (2007) proposed presenting a video replay strategy using a secondary screen to quickly provide situation awareness after prolonged times of robot neglect.

##### Audio and speech

Our ability to localize acoustic sources and apply selective attention to one acoustic stream out of many, even at a distance, makes the audio modality popular for communicating failures. As such, many mobile robots use audio and speech to communicate robotic failures. Some use simple audio tones to gather user attention (e.g., Brooks, 2017), whereas others communicate failure using more complicated speech, such as Jibo^2^ and the robot in Schütte et al. (2017). Cha et al. (2015) found that people perceived robots speaking conversationally as more capable than those that could only maintain a functional level of speech. However, this changed when the robot made an error—after an error, robots with conversational speech were perceived as less capable than those with functional speech. This effect is similar to equivalent research in HCI (Weinstock et al., 2012) that found that when a visually aesthetic user interface errors, the error lowers perceptions of satisfaction, human automation cooperation and trust more than when a non-visually aesthetic interface errors. Several researchers suggest to use verbal communication cautiously since dialogue can lead to biased perceptions of the robot's capabilities (Fong et al., 2003; Cha et al., 2015). Simpler audio signals can be used to signal the existance of a problem, however, they cannot effectively explain the cause of error.

##### Modality comparisons

Very few studies assessed the benefits of different modalities for communicating failures in HRIs. Cha et al. (2016) evaluated a robot which utilized both light and sound of varying levels of urgency to request help from bystanders when it experienced difficulty. Results indicated that participants interpreted light and sound signals differently: sound alerted the user that the robot needed help and the light indicated the level of urgency of the help request. Moreover, participants preferred a more attention-grabbing signal when the urgency of the request was high, and when the urgency of the request was lower, they preferred the robot to take into account the participant's level of availability by utilizing greetings and being more polite.

Brooks (2017) compared between a designated smartphone application and a light-and-button based interface in their ability to help inexperienced users better detect and solve failures while performing a secondary task. Unlike the previous example, which used an indicator to help users detect robot requests, this example focused also on its ability to help users solve errors. Results indicated that participants were able to obtain information about the robots, identify solutions to problems and allocate their time more appropriately using the app.

Further studies from the warning literature provide insight regarding how to create comprehensible warnings. Warnings presented in more than one modality generally facilitate better comprehension than those presented in a single modality (Wogalter, 2006a). While there is conflicting evidence of whether written text or speech are better for comprehending language-based warnings (Mayer, 2002; Wogalter, 2006b), reading language allows people to review the material and tends to be faster, so it may be more appropriate for long or complex messages. In contrast, shorter, less complex messages have a greater impact when presented auditorily than visually, and are generally better for switching attention (Wogalter, 2006a). A short auditory warning that directs the users' attention to more detailed information could be used to capture attention while facilitating the processing of more complex information (Wogalter, 2006a).

### Perception and comprehension of failures

#### Attention switch and maintenance

For a failure event to influence user behavior, attention must be switched to it for the user to perceive the information (Wogalter, 2006a). Moreover, attention must be maintained by users to perform desired behaviors properly and avoid certain types of human errors, such as slips (Reason, 1990). The conditions under which a person shifts their attention can be used to guide the design of robotic failure indicators. Sudden changes in the environment [e.g., change in luminance (Theeuwes, 1995), motion onset (Abrams and Christ, 2003), and abrupt appearance or disappearance of stimuli (Pratt and McAuliffe, 2001)] or the robot's behavior (Okada et al., 2003; Sato et al., 2007) could be used to quickly and involuntarily shift people's attention to urgent failure situations or to cue users to attend to information elsewhere. These involuntary shifts of attention tend to be brief (Buschman and Miller, 2010), and are dependent upon users' expectations (Posner et al., 1978; Folk et al., 1992). In contrast, long term exposure to a warning could make it unable to attract attention at later times (“inhibition of return”; Posner and Cohen, 1984; Klein, 2000), so the use of permanent cues must be considered carefully.

Voluntary shifts of attention can be sustained for longer periods of time (Welsh et al., 2009) and can result from a wider variety of stimuli (Sears and Jacko, 2009), allowing more freedom in the design of failure indicators. Various factors affect people's ability to identify and attend to a specific stimulus, including the degree of similarity to other items in the environment (von Grünau et al., 1994; Gorbunova, 2017), interest (Renninger and Wozniak, 1985), temporal and physical location of warnings (Frantz and Rhoades, 1993; Wogalter et al., 1995), the task (Welsh et al., 2009), age (Yamaguchi et al., 1995), and practice (Feinstein et al., 1994). This emphasizes the importance of taking contextual factors into consideration when designing warnings for failure. Fischer et al. (2014) found that verbal greetings attracted attention better than simpler audio signals, but they did not improve the likelihood of the person to perform the robot's request.

The design of a warning should be guided by the response required from the user (stimuli-response compatibility; Sears and Jacko, 2009). For example, reaction time is lower when people are asked to respond vocally to an auditory stimulus or with motion to a spatial attribute (Wang and Proctor, 1996). Spatial correspondence (Fitts and Seeger, 1953; Fitts and Deininger, 1954; Reeve and Proctor, 1990), similarity (Kornblum et al., 1990), and logical relations (rules) (Duncan, 1978) between the stimulus and response sets have all been shown to improve stimulus-response compatibility. Since it is not always clear in which circumstances compatibility effects are going to occur (Proctor and Vu, 2009), designers need to repeatedly test warnings on users, particularly for urgent failures.

A robot's warning can be noticed yet fail to maintain attention long enough for the user to extract meaning from it (Wogalter, 2006a). The required duration of attention maintenance has been shown to rely on the channel of communication as well as on the complexity and form of the content (Wogalter, 2006a). Generally speaking, if a warning contains too much information, is too hard to read, or the relevance of the information is low or unclear, people may decide it is too much effort, lose interest and direct their attention elsewhere (Wogalter, 2006a). Moreover, as felt involvement with product information increases, consumers have been shown to spend more time attending to the information (Celsi and Olson, 1988). Combining pictures with written or spoken text has been shown to increase attention to information in comparison to text alone (Houts et al., 2006). Visual warnings with organized information groupings and generous white space are more likely to hold attention than a single block of text (Wogalter and Vigilante, 2006). The use of humor has also been shown as an effective way to gain and maintain attention (Weinberger and Gulas, 1992). These strategies could be used in the design of warnings to promote compliance.

#### Comprehension and memory

Users must be able to understand the meaning of a failing robot's symptoms or the warning it provides to understand what the failure is and how to react. During the comprehension process, incoming perceptual inputs that have passed attentional filters are connected to past experiences or knowledge to construct an understanding of the event (Harris et al., 2006). This continuing interaction of comprehension and memory is important to understanding what may influence a person's ability to relate erroneous behavior to “normal” robotic behavior, to comprehend the meaning of a failure indicator and to resolve robotic failures.

Characteristics of memory have several implications for robotic failure situations. While people can remember large amounts of information over their lifetime, only a small portion is available to them at any given time for processing (Bettman, 1979; Lang, 2000). As a result, memories and knowledge may not become available without an external cue (Wogalter, 2006a), and those that are readily available may quickly become unavailable due to interference or decay (Proctor and Vu, 2009). This emphasizes the importance of considering external factors, such as user tasks and bystanders, and of providing informative cues to help the user recall and resolve a failure.

In failure-handling situations, recall and comprehension of relevant information (warnings, robotic commands, and possible solutions) could be made easier by exploring influential factors. Studies indicate that it is easier to recall information that is visual (Paivio and Csapo, 1973), concrete (Butter, 1970; Sheehan and Antrobus, 1972), repeated (Kintsch et al., 1975), specific (Mani and Johnson-Laird, 1982), personal (Van Lancker, 1991), novel (Kishiyama and Yonelinas, 2003), typical (Reeve and Aggleton, 1998), humorous (Schmidt, 1994; Summerfelt et al., 2010; Carlson, 2011) and self-generated (Wheeler and Gabbert, 2017). The likelihood a retrieval cue leads to recollection depends on the similarity between the features encoded initially and those provided by the retrieval cue, distinguishability from other cues, and association with the newly learned information (Wheeler and Gabbert, 2017). Storing information to memory seems to depend on deep processing of the meaning of new material, determined by the degree to which one understands the information to form meaningful associations and elaborations with existing knowledge (Bower, 2000), as well as on arousal (Butter, 1970) and individual differences (Verhaeghen and Marcoen, 1996) [e.g., age (Anderson et al., 2000), mood (Bower et al., 1978)]. Various techniques have been developed to improve recall and storage from and to memory (e.g., Bower, 1970a,b; Ritchie and Karge, 1996; Gobet et al., 2001). Such techniques could be used by robot designers to help select appropriate cues that help users recall information that is relevant to the failure.

Comprehension has been shown to be influenced by background knowledge (Tannenbaum et al., 2006), wording (Kintsch et al., 1975), typographic design (Frase and Schwartz, 1979), personality (Sadeghi et al., 2012), felt involvement (Celsi and Olson, 1988), motivation (Sideridis et al., 2006), expectations (Haberlandt, 1982), training (Dewitz et al., 1987), experience (Macias, 2003), level of automation (Carmody and Gluckman, 1993), interface design (Canham and Hegarty, 2010), workload (Perry et al., 2008) and stress level (Perry et al., 2008). One common way to classify a person's level of comprehension is by evaluating their Situation Awareness (SA) (Endsley, 1988). Drury et al. (2003) defined components of situation awareness that are relevant to HRI: (1) awareness of the locations, identities, activities, states, and surroundings of the robot and fellow human collaborators, (2) awareness of the robot's knowledge of the human's commands and any human constraints, (3) awareness of the knowledge that the robots have of the activities and plans of other robots, and (4) awareness of the overall goals of the joint human-robot activities and progress toward the goal. They then related these types of awareness to critical incidents at an urban search and rescue competition in which the operator or robot encountered a problem, and found that all critical incidents resulted from awareness violations (Drury et al., 2003). Techniques that improve situation awareness could be used by robot designers to help prevent various types of failures.

#### Beliefs and attitudes

At this stage of processing, the comprehended information merges with existing beliefs and attitudes. A mental model can be a useful concept for understanding this process. As the user interacts with the robot, they receive feedback from the system and the environment that allows them to develop a representation (a mental model) of how they believe the system behaves for a given task. These representations lead to expectations, which in turn direct perception and behavior (Stanton, 2009). Studies in the field of HCI found that users infer models that are consistent with their experiences, even when there is lack of evidence that supports their assumptions (Payne, 2009). Moreover, instead of developing unified models, they develop separate beliefs about parts of the system, processes, or behaviors that are not necessarily complementary (Payne, 1991). While incorrect mental models can lead to difficulties in problem solving, the use of appropriate mental models can help people learn, remember and execute procedures faster (Kieras and Bovair, 1984). Mental models can also explain human errors: if action is directed by mental models, then the selection of inappropriate models or erroneous activation of appropriate models will lead to errors (Norman, 1981). Designers can increase the usability of a robotic interface for handling failures using metaphors that promote the use of applicable mental models and by correcting inappropriate mental models through feedback.

In the HRI literature, mistakes made by robots influence how the robot is perceived. Failures reduce robots' perceived sincerity (Gompei and Umemuro, 2015), competence (Cha et al., 2015; Salem et al., 2015; Ragni et al., 2016), reliability (Salem et al., 2015; Ragni et al., 2016), understandability (Salem et al., 2015), trustworthiness (De Visser and Parasuraman, 2011; Desai et al., 2013; Salem et al., 2015; Law et al., 2017), intelligence (Takayama et al., 2011; Bajones et al., 2016; Ragni et al., 2016), and likeability (Bajones et al., 2016; Mirnig et al., 2017), and increase perceived familiarity (Gompei and Umemuro, 2015). In Kahn et al. (2012), participants who interacted with a humanoid robot that incorrectly assessed their performance perceived the robot as having emotional and social attributes. Research is inconclusive regarding the effect of failures on the robot's perceived anthropomorphism. Salem et al. (2013) found that errors made robots seem more human, whereas Salem et al. (2015) found that it made robots seem less human. Mirnig et al. (2017), in contrast, did not find differences in people's ratings of the robot's anthropomorphism and perceived intelligence. These differences may be a result of the different robots used, or the different interaction contexts (task, environment).

User perceptions of the robot in a failure situation seem to be influenced by a number of factors. In contrast to Salem et al. (2015), which found that failure reduced perceived reliability, technical competence, understandability, and trustworthiness of a home-care assistant robot, the manufacturing robot in Sarkar et al. (2017) was perceived in a similar manner regardless whether it was faulty or not. According to Sarkar et al. (2017), these differences may stem from the type of failures (Sarkar et al., 2017 involved subtle interaction failures, whereas Salem et al., 2015 produced physical failures with potentially irreversible consequences), or the nature of the experimental task (the industrial context in Sarkar et al., 2017 compared to a more “social” setting in Salem et al., 2015). Rossi et al. (2017a) found that errors with severe consequences lead to greater loss of trust in the robot. Furthermore, user perceptions of the robot in a failure situation may depend on attribution of the cause of failure—in an online survey (van der Woerdt and Haselager, 2017), participants were shown a video portraying a NAO robot failing a task either due to lack of ability or lack of effort. In case of failure, participants attributed more agency to the robot that displayed lack of effort compared to videos in which it displayed lack of ability. The timing of failure also seems to influence how the failure affects perceptions of the robot. Gompei and Umemuro (2015) investigated the effect of a failure's timing: when the robot made speech errors on the first day of contact, the robot's familiarity score did not change; when the robot made its first speech error on the second day of contact, the robots' familiarity score moderately improved as a result of the error. Similarly, Lucas et al. (2017, 2018) found that errors that occur later in a robot's dialogue, particularly after a period of good performance, reduce the robot's persuasiveness.

While robotic failures have been shown to reduce the perceived trustworthiness of robots (De Visser and Parasuraman, 2011; Hancock et al., 2011; Desai et al., 2013; Salem et al., 2015; Law et al., 2017), users' compliance with robot instructions may not be affected. Robinette et al. (2016, 2017) evaluated whether people will trust and follow the directions of a faulty robot in emergency evacuee scenarios. Results showed that the vast majority of participants followed the instructions of the robot despite erraneous behaviors. In line with this finding, Salem et al. (2015) found that while the robot's erratic behavior affected its perceived reliability and trustworthiness, it did not impact participants' willingness to comply with its instructions, even when the requests were unusual. Severity of the outcome affected compliance with robot requests (Salem et al., 2015). Similar effects were found by Tokushige et al. (2017) as a result of unexpected recommendations.

While there are some indicators that people may prefer predictable behavior in robots (Mubin and Bartneck, 2015), others suggest that people feel more engaged by unpredictable behavior (Short et al., 2010; Fink et al., 2012; Lemaignan et al., 2015; Law et al., 2017). Various studies seem to suggest that failures can be a source of pleasurable interaction with robots (Bainbridge et al., 2008; Yasuda and Matsumoto, 2013; Gompei and Umemuro, 2015; Ragni et al., 2016; Mirnig et al., 2017). In a study by Ragni et al. (2016) despite the faulty robot being rated worse than the error-free robot, participants reported greater enjoyment when the robot made errors. Similarly, Mirnig et al. (2017) found that participants liked faulty robots better than robots that interacted flawlessly. Annotations of video data showed that gaze shifts, smiling and laughter are typical reactions to unexpected robot behavior. While these studies provide insight regarding reactions to robotic failures, the non-criticality of the errors coupled with low personal relevance to the participants may have impacted results.

Desai et al. (2013) investigated the influence of varying reliability on real-time trust and found that periods of low reliability earlier during the interaction have a more negative impact on overall trust than periods of low reliability later in the interaction. In contrast, a preliminary study by Desai et al. (2012) found that people trust a robot less when reliability drops occurred late or in the middle of runs. Within the broader human-automation literature there is certain agreement that trust depends on the timing, consequence, and expectations associated with failures of the automation (Lee and See, 2004).

### Solving failures

#### Motivation

Solving a robotic failure requires the user to be motivated to solve the problem. Even if the users are not capable of solving the failure themselves, they need to be motivated enough to inform other agents of the problem (such as a caregiver or a technician) in order for it to be addressed. While some problems may significantly impact users, motivating them implicitly, other failures may not be sufficient to motivate them enough to solve the problem, particularly if the interface is hard to understand or operate. Thus, creating successful failure-handling solutions requires skills in motivating and persuading people. Captology, the study of persuasive technologies is a relatively new endeavor in HRI (see Siegel, 2008; Ham and Spahn, 2015). Research has explored effect of a robot's physical presence (Kidd and Breazeal, 2004; Shinozawa et al., 2005; Bainbridge et al., 2008), touch and gesture (Shiomi et al., 2010; Ham et al., 2011; Nakagawa et al., 2011; Chidambaram et al., 2012; Baroni et al., 2014), gazing (Ham et al., 2011), robot and user gender (Siegel, 2008; Nakagawa et al., 2011), vocal cues (Chidambaram et al., 2012; Baroni et al., 2014), interpersonal distance (Siegel, 2008), reciprocity (Lee and Liang, 2016), conversational errors (Lucas et al., 2018), agency (Ham and Midden, 2011), and perceived autonomy (Siegel, 2008) on persuasive effects. However, none of these studies focused specifically on the influence of motivation in solving robotic failures.

Robots are sometimes viewed as tools, and other times viewed more as social actors (Breazeal, 2004). According to Fogg et al. (2009), there is a difference in how computers can be used to persuade, depending on whether they are viewed as a tool or social actor. Computers as tools can persuade by providing tailored information, triggering decision making, increasing self-efficacy, and guiding people through a process. In contrast, computers as social actors can persuade people by providing social support via praise or criticism, modeling behaviors or attitudes, and leveraging social rules (e.g., turn taking, politeness norms, praise and reciprocity).

#### Decision-making

Once individuals have perceived the failure symptoms and/or warnings, comprehended them, formed beliefs and attitudes regarding the situation, and gained enough motivation to solve the issues, they must decide what can be done to solve the failure. Most problems are well beyond the capacity of comprehension to be solved optimally. Reaction time typically increases with the number of stimulus-response alternatives (the Hick-Hyman law; Hick, 1952; Hyman, 1953). Consequently, for problem solving to be effective in a robotic failure situation, search must be constrained to a limited number of possible solutions or approaches (Proctor and Vu, 2009).

A common way novice users constrain search in situations of uncertainty is to use heuristics (Tversky and Kahneman, 1974). Research demonstrates that our judgements are based on the subset of relevant information most accessible in memory, and that we rarely retrieve all relevant information (Bodenhausen and Wyer, 1987; Schwarz, 1998). One particularly common strategy is “satisficing” (Simon, 1956), which refers to searching through available alternatives and choosing the first that meets some minimum acceptable threshold. Some other examples include (but are not limited to) representativeness (Tversky and Kahneman, 1973), availability (Tversky and Kahneman, 1973), and adjustment (Epley and Gilovich, 2006) heuristics. The problem with using heuristics is that they often lead to cognitive biases, which influence the quality of the decision. Many biases in human decision making have been discovered (Croskerry, 2003) [e.g., the framing effect (Tversky and Kahneman, 1981), confirmation bias (Nickerson, 1998), and overconfidence effect (Dunning et al., 1990)]. Consequently, people generally make nonoptimal decisions.

Various efforts have been made to improve and debias decision making, which could be implemented to better support users during robotic failure situations. Three general approaches have been suggested and shown to produce positive results (Morewedge et al., 2015): (1) recalibrating incentives to reward healthy behavior, (2) optimizing how choice options are presented and obtained, and (3) debiasing training interventions. Small changes in presentation and elicitation of choices are particularly effective, cheap and easy to implement, taking many forms such as information framing (Levin and Gaeth, 1988; Larrick and Soll, 2008) and default selection (Johnson and Goldstein, 2003; Chapman et al., 2010). These recommendations, alongside additional strategies (e.g., Croskerry, 2003), could be used to help facilitate the design of failure-management interfaces for robots to improve the problem-solving abilities of untrained users.

#### Act

This stage of processing refers to both the execution of the person's decision regarding how to respond to the robotic failure, as well as automatic behaviors that are triggered without maintaining attention. People seem to have various predictable behavioral responses to robotic failures that can be used by robots to identify when a failure has occurred. Failure has been shown to influence users' gaze patterns (Gehle et al., 2015; Hayes et al., 2016; Mirnig et al., 2017), facial expressions (Hayes et al., 2016; Mirnig et al., 2017), head movements (Hayes et al., 2016; Mirnig et al., 2017; Trung et al., 2017), body movements (Mirnig et al., 2017; Trung et al., 2017), and verbal communication (Gieselmann, 2006; Giuliani et al., 2015). Gieselmann (2006) found that indicators for errors in human-robot conversation included sudden changes of the current dialogue topic, indicating non-understanding by asking unspecific questions, asking for additional information and repeating the previous question. Additional indicators used to detect errors in spoken human-robot dialogues include people being silent, asking for help, repeating central elements or asking the robot repeatedly for the same information, saying things that are inconsistent with the current discourse or with the robot's expectations, trying to correct a preceeding utterance, hyperarticulating speech, or asking for something they know the robot cannot do, such as making coffee (Gieselmann and Ostendorf, 2007).

Giuliani et al. (2015) and Mirnig et al. (2015) analyzed video data showing social HRIs in which the robot unintentionally made an error. Results indicated that in erraneous situations, participants often used head movements, smiled, raised eyebrows, and looked back and forth between the robot and experimenter or a group member if present. Moreover, the type of error (social norm violation or technical failure) as well as the presence of other people seemed to impact people's reactions to the failure. More specifically, during social norm violations, participants spoke more, were more likely to look back and forth between the robot and objects in front of them and say task-related sentences to the robot than during technical failures. When no experimenter or person was visible, participants used fewer non-verbal social signals (e.g., smiling, nodding, and head shaking), and more often shifted their gaze between the robot's hand, the robot's head, and other objects in front of them than when the experimenter was visible, or when interacting in groups with the robot. The presence and response speed of these social signals were dependent on the type of error made and the type of task the robot was performing.

There is also reason to believe that the modality of the failure influences people's reactions. Short et al. (2010) investigated people's reactions to playing rock–paper–scissors with a humanoid robot that either played fair, cheated through action by changing the selected hand gesture or cheated verbally by declaring a different hand gesture than the one used. Results indicated that participants showed more verbal social signals to the robot that cheated. Interestingly, verbal cheating was perceived as malfunctions, often leading to reactions of confusion, whereas cheating through action was perceived as deliberate cheating, leading to more exaggerated reactions, showing surprise, amusement, and occasionally anger.

### Contextual factors

#### Receiver

The receiver is the person(s) or target audience whom witness the warning or symptom, typically the user. Personal attributes of robot users have been shown to affect all stages of information processing, and in turn, the stage of information processing influences the users' experiences and behaviors. Contributing factors surveyed include the user's attitudes and beliefs, interest, practice and training, experience, background knowledge, workload, stress level, situation awareness, mental model, and gender.

#### Environment and other agents

External stimuli from the environment compete for the receiver's limited attention and comprehension resources, limiting information processing. For instance, a friend saying “Hi” when the robot is trying to indicate that the motors stopped working could prevent the user from attending to a visual warning. A noisy environment may cause the user not to hear the robot's low battery beep, or not to be able to concentrate enough to lead it back to its charger. In some cases, this could be an advantage: social norm violations, for instance, could be missed and therefore not negatively influence the interaction. The individual may act on the environment and change it, so there is a bilateral relationship between the environment and the stages of information processing. In situations where the user does not have the know-how, ability or the tools to fix the problem, the involvement of other agents may be necessary to solve the failure.

#### Task

Task refers to attributes of either the robot's task, the person's task, or a joint task to be completed together. From the literature, it is evident that the task a person is performing can compete for their limited attention and comprehension resources and by doing so, impact the stages of information processing. In turn, cognitive resources devoted to the failure have an impact on the task: an increase in automation during failure condition reduces operator performance (the “lumberjack analogy”; Sebok and Wickens, 2017). Several studies seem to indicate that task performance is significantly influenced by robotic failures. In Ragni et al. (2016), participants competed against a robot in reasoning and memory tasks where the robot either performed with or without errors. Results indicated that task performance was significantly lower in the faulty robot condition. Similarly, in Desai et al. (2012), drops in reliability were shown to affect participants' self-assessments of performance. Salem et al. (2013) evaluated whether participants who were presented with incongruent multimodal instructions by the robot performed worse at their task than those who were presented with unimodal or congruent multimodal information by the robot and found that incongruent coverbal gesturing reduced task performance. One contrasting account is the manufacturing scenario described in Sarkar et al. (2017), where a physical object was assembled and then disassembled under regular and error conditions. In this scenario, faults did not affect the successful completion of a manufacturing task. The authors proposed that these results may be because the types of failures they implemented (missing an action and/or giving the wrong instructions) did not impede the possibility of a successful manufacturing outcome.

### Mitigation strategies

Various mitigation strategies can be attempted both by the user and robot in order to prevent and handle the negative influences of failure. Mitigation strategies could be applied in any stage of information processing. The stage of processing, in turn, affects the effectiveness of the mitigation strategy applied. The following sections discuss the various strategies that have been implemented to mitigate the negative effects of failure in HRI.

#### Setting expectations

Giving the user advance notice regarding potential failures influences how they respond to subsequent failures. This is consistent with studies that found that robotic errors have a stronger negative effect after a period of good performance (Lucas et al., 2018). One online study by Lee et al. (2010) found that setting expectations by forewarning participants of the abilities of the robot improved evaluations of the robot and judgments of the quality of the service. Providing options helped increase people's willingness to use the robotic service again after failure, however was not particularly effective in improving perceptions of the robot (Lee et al., 2010). Additional studies found that providing confidence feedback on the robot's performance encourages better control allocation without affecting user trust (Desai et al., 2013; Kaniarasu et al., 2013).

#### Communicating properly

Several researchers have evaluated the impact of politeness strategies, such as apologizing (Lee et al., 2010; Peltason and Wrede, 2011) or expressing regret (Hamacher, 2015), on human-robot error interactions. When robots employ these strategies, perceptions of robots and responses to disagreement are improved (Takayama et al., 2009; Torrey, 2009). In Hamacher et al. (2016) apologizing, expressing regret and expressing reparation lead to similar trust ratings as a non-failing robot.

Various repair strategies have been used to help robots gracefully recover from verbal misunderstandings and speech errors (Gieselmann, 2006). *Achievement strategies* involve explaining the meaning of an utterance, e.g., paraphrasing, restructuring the sentence, repetition, and asking for help. *Functional reduction strategies* involve replacing the original intention by a different, simpler one, for instance, telling the robot to go to the kitchen instead of telling it to pick up the cup in the kitchen. *Formal reduction strategies* involve simplifying the grammar or the vocabulary used, and *ratification* involves confirming or repeating the last utterance made (e.g., “yes, I asked you to press the green button”). Gieselmann (2006) evaluated the use of these strategies in a domestic HRI scenario, and found that the most common error recovery strategies were achievement strategies and functional reduction strategies.

There is little research evaluating what information should be communicated to help users cope with robotic failure situations. One research study (Cameron et al., 2016a) proposed a method to evaluate whether a robot should respond to an error with (1) simple instructions for the user to follow (e.g., “Follow me back to the lift”); (2) competency-oriented statements that emphasize the robot's abilities, the current situation, and goal (e.g., “That sign said we are on C floor and we need to go to B floor. Follow me back to the lift”); (3) inclusion of apology-oriented statements that emphasize attempts to relate to users but do not indicate competency (e.g., “Sorry about the error; we all make mistakes sometimes. Follow me back to the lift”); or (4) inclusion of both the competency- and apology-oriented statements. However, to the best of our knowledge, the results of this experiment have yet to be published. Other studies proposed communicating the cause of error with varying degrees of success. One experiment found that having the robot place blame for a failure reduced user trust (Kaniarasu and Steinfeld, 2014). Another study found that attributing blame to the user led people to feel less comfortable with the robot, perceiving it as less friendly and competent, even when the person was likely aware that they were the source of problem (Groom et al., 2010). Kim and Hinds (2006) found that providing the cause of failure could facilitate more accurate blame-attribution as long as the robots' explanation correlated to the background knowledge of participants. If not, providing the cause decreased people's perceived understanding of the system. Kwon et al. (2018) proposed expressing physical limitations through motions that communicate what the robot attempted to accomplish and why it was unable to accomplish it. The use of these motions was found to increase positive evaluations of the robot and willingness to collaborate.

It also seems to be important for the robot to produce appropriate verbal and non-verbal responses to an error. One study evaluated how a robot's gaze behavior (no gaze, looking at the other, looking down, and looking away) during mistakes change people's impressions (Shiomi et al., 2013). Experimental results showed that “looking at the other” outperformed different gaze behaviors, communicating degrees of perceived apologetics and friendliness and providing more reflection. Takayama et al. (2011) found that showing a goal-oriented reaction to a task outcome (i.e., disappointment in response to failure and happiness in response to success) made the robot appear smarter than when it did not react, regardless of whether the robot succeeded or failed in the task. Hamacher et al. (2016) found that demonstrating appropriate emotions and awareness of error (e.g., regret or enthusiasm) significantly tempers dissatisfaction with a robot's erroneous behavior and improves trust. Gieselmann (2006) evaluated user reactions to different robot error indicators and found that people preferred the robot asking a specific question to obtain additional information when it didn't understand their utterance. Indicating non-understanding with unspecific questions left users confused, since they did not know what the robot did not understand, hindering their ability to solve the error.

#### Asking for help

Several researchers proposed having robots request help from a human partner when they encounter an error (Ross et al., 2004; Hüttenrauch and Severinson-Eklundh, 2006; Rosenthal et al., 2012; Yasuda and Matsumoto, 2013; Knepper et al., 2015; Bajones et al., 2016). This strategy is computationally less expensive than re-planning, however it is not always applicable (e.g., when the people around do not have the ability or knowledge to help the robot solve the problem). In situations where it is applicable, asking for help can lead to negative experiences (e.g., Mutlu and Forlizzi, 2008) and can be very expensive in terms of monitoring time and cognitive load (Rosenthal et al., 2012). In such cases, it seems the way the robot asks for help matters. Knepper et al. (2015) developed a system that allows a robot to specify the kind of help that is needed in a way that removes as much ambiguity as possible. Users reported that they felt the system was more effective at communicating needs than other tested methods; preferring the precise requests over general phrasings. Moreover, the system improved the subjective evaluation of the robot and the speed and accuracy of human intervention when the robot experienced a problem. Maintaining polite communication also seems to matter: Yasuda and Matsumoto (2013) experimented with a robotic trashcan that spilled garbage, asked a person to pick up the trash for it and then “bowed” in appreciation. Most people found the experience to be positive, despite the spilled garbage and request for help. Another study found that participants who saw the robot stating its limitations before asking for help reported liking the robot more than those who saw control statements (Cameron et al., 2016b).

Rosenthal et al. (2012) sought to understand the willingness and availability of occupants to help a service robot. In their study, a robot visited different offices at different times of day, with different types of requests, and recorded willingness to provide help and the duration of that help. Participants were equally willing to help with all types of requests. Interestingly, willingness to help was not affected by the length of time the question took to answer nor the incentives the occupants received. In a related study, Srinivasan and Takayama (2016) evaluated factors that influence people's behavioral willingness to help a robot, finding that it depends on the robot's social role (peer or assistant), familiarity (new vs. 10 years experience), level of autonomy (autonomous or teleoperated), politeness strategy (direct request, positive politeness, negative politeness, or indirect request), and size of request (small or large). More specifically, people were more willing to help a peer robot that made smaller requests (i.e. that require less effort to fulfill), was more familiar, and used a positive politeness strategy (attended to the listener's wants, conveyed liking, and made the listener feel good about themselves). Moreover, Participants were nearly 50% quicker to help the robot when they believed that it was behaving autonomously rather than being teleoperated by a person.

The aforementioned work largely deals with preventing failures related to limited capabilities or missing information by proactively requesting help. However, some failures cannot be foreseen in advance and may not be included in the robot's planner (i.e., Black Swans; Sebok and Wickens, 2017). Bajones et al. (2016) performed a multi-user Wizard-of-Oz experiment in which they asked participants to help a malfunctioning robot restore the interaction flow after an error occurred. Results indicated that all 38 participants were willing to help the robot with repeated failure situations, regardless of the role they were given in the interaction (“director” or “builder”). Moreover, they found that the person who gave the last command was more likely to help, followed by the person who was closer. Malfunctions that could be actively fixed by the participants did not negatively impact perceived intelligence and likability ratings of the robot.

#### Mix and match

Researchers have combined mitigation strategies in order to increase their effect. Spexard et al. (2008) implemented a model that decided on the best strategy based on the initiative taker and the solution provider of an error. Hardware defects caused the robot to inform the user of the reason why it could not move and ask for help, mode confusion or the robot behaving unexpectedly caused it to prompt the user to reset the system, software failures caused the robot to inform the user about the break-down, asking them to contact a technician. Using these help strategies, all participants successfully coped with the problem without external help.

There is very little work on comparing different failure recovery strategies. One exception is Lee et al. (2010), which investigated people's reactions to different recovery methods (apologies, compensation, and options for the user) in an online survey. All the recovery strategies increased positive ratings of the robot's politeness, however, only the apology strategy was effective in making the robot seem more competent, and in making the participants feel closer to the robot and liking it more. The compensation strategy was most effective in increasing perception of satisfaction with the service, but less effective than the apology and option strategies in increasing their perceived willingness to use the service again. The results also suggest that tailoring the recovery strategy to people's orientation to services is important—people with a relational orientation responded particularly well to an apology whereas those with a more utilitarian orientation responded better to compensation. Moreover, apologies were shown to be better for people who treated the robot more like an agent, while compensation was better for people who treating it like a tool. Another study that investigated different failure recovery strategies is Engelhardt and Hansson (2017), which compared between: “ignore” (the robot ignores that a failure has occurred and moves on with the task), “apology” (the robot apologizes for failing and moves on) and “problem solving” (the robot tries to solve the problem with the help of the human). Results showed that the apology strategy scored the lowest on likeability and perceived intelligence, and that the ignore strategy lead to better perceptions of perceived intelligence and animacy. Problem-solving clearly minimized the negative effects of failure better than apologizing, but the “ignore” condition often scored at least as well as problem-solving.

Several theories have been suggested to explain successful mitigation strategies. According to Booth (1991), whether system errors are helpful or disruptive depends on (i) the ease with which the user can recover from an error; and (ii) the extent to which the system provides cues or features that productively direct the user toward a more appropriate understanding. In line with this theory, Brooks et al. (2016) argued that providing *human support* (providing information that supports or improves the user's situation awareness with respect to the failure and the status of the task being performed) or *task support* (helping the user complete the task they wanted to accomplish) will mitigate negative effects caused by failure; and that combining the two techniques should minimize problems without negative side effects. Moreover, they hypothesized that recovery strategies which reduce the negative effects of a failure will also increase the likelihood of users wanting to use the system again. To test these hypotheses, they conducted two between-subjects survey studies (Brooks et al., 2016). Results indicated that human support was better correlated to whether the information conveyed could be used by the person to affect the outcome of the situation. Task support, as well as a combination of task support and human support, significantly improved people's reaction to failure in all but one scenario. Recovery strategies that reduced the negative effects of a failure were shown to increase the likelihood of users to want to use the system again.

## Discussion

The majority of published works on robotic failures focus on technical aspects of making the robots more reliable. Few studies have actively worked toward making failure-handling user friendly, however the growing number of publications on the topic seems to indicate an increase in interest. Successful failure-handling strategies that enable untrained users to quickly and easily identify and solve failures require a holistic approach to design and development. The technical knowledge of hardware and software must be integrated with cognitive aspects of information processing, psychological knowledge of interaction dynamics and domain-specific knowledge of the user, the robot, the target application, and the environment. To achieve this, additional research is essential. By combining insights from a large variety of fields into a single framework, RF-HIP can be used to guide these discussions, and provides an initial hypothesis regarding how people might process symptoms and warnings in situations of robotic failure. In a similar manner to how C-HIP supports the design of new warnings and alerts, the stages of processing could be used to help determine why a particular approach of handling failure is successful while another is unsuccessful; leading to informed design tools and guidelines that facilitate the development of robot interactions that enable untrained users to quickly and easily identify and act upon failures.

Several gaps in the literature have become evident as a result of this analysis. First, it seems that most efforts have been focused on how failures influence user perceptions of the robot and user behavior, looking primarily at cause and effect. Little work has been done on evaluating how a robot should communicate that an error has occurred. Almost no work has been done to understand the underlying cognitive, psychological, and social determinants behind these relationships and how they may impact selection of mitigation strategies. Second, there seems to be a great asymmetry in the types of failures being studied and subsequent failure-handling strategies proposed: while there is a lot of emphasis on recovery strategies to cope with technical failures, there aren't any strategies to cope with recovery from human errors—equivalent to cancel or undo in HCI. Moreover, social-environmental considerations such as the work environment, group-level judgement, and organizational flaws have not been taken into consideration. Third, the importance of motivation to how people perceive, comprehend and solve robotic failures seems to be lost in the literature—studies typically evaluate people in unnatural settings, using tasks that are low in personal relevance. As a result, the ecological validity of most of the studies is low. It would be interesting to evaluate how motivation might influence responses in a more natural setting, when participants have a real stake in whether the robot will succeed or fail. Fourth, the failure attributes identified (functional severity, social severity, relevance, frequency, condition and symptoms) have not received almost any consideration in the HRI literature in terms of how they influence the way in which the failure should be communicated, the HRI, and the selection of mitigation strategies. For the most part, these attributes are unexplored territory and require targeted assessment. Lastly, since most studies used indoor, single-person environments, the effects of various aspects of the environment (e.g., other agents, weather, lighting, size of space) on perceptions of failures and preferences of communication and mitigation strategies remain unknown.

Another challenge the robotics community is facing in failure-handling is benchmarking and comparability. The wide variety of robotic implementations, evaluation environments and measures, coupled with lack of consistency on which implementation and evaluation details are reported in scientific publications, make it difficult and nearly impossible to compare subjective and objective performance metrics from different failure-handling studies. We are unaware of any frameworks that specify how all the contextual considerations identified in this paper should affect robot behavior in order to produce a pleasurable experience. Development of such frameworks are likely going to come from comparing and combining different implementation methods with insights from a wide variety of user studies. A common benchmark must be crafted for a set of robots, tasks, environments, and conditions. Consistent subjective measures and batteries of questionnaires along with clear quantitative evaluation measures must also be defined.

From the literature survey it is evident that many aspects remain to be studied in the field of user-centered failure handling, making this an exciting time to be active in the field. The importance of studying cognitive considerations that critically influence naive users' ability to detect and solve robot failures is evident. While the current paper proposes how failure warnings and symptoms may be perceived by people, the specifics of the proposed framework must be thoroughly tested and verified. Moreover, whether the RF-HIP model can be used to predict the impact of various forms of robot design on a users' ability to handle failures is still to be determined. Hopefully, this review provides a good starting point for discussing what needs to be done in order to develop robot interactions that enable untrained users to quickly and easily identify and solve failures.

## Author contributions

SH is the first author of this publication and main contributor. TO-G is her Ph.D. advisor.

### Conflict of interest statement

The authors declare that the research was conducted in the absence of any commercial or financial relationships that could be construed as a potential conflict of interest.

## Footnotes

^1^“Status Lights,” *Neato Robotics* (2017). Available online at: https://support.neatorobotics.com/hc/en-us/articles/225370027-Status-Lights (Accessed December 14, 2017).

^2^“Hey! I'm Jibo.” Available online at: https://www.jibo.com/. [Accessed: 14-Dec-2017].

^3^“Jibo - Error messages.” Available online at: https://support.jibo.com/jibo/articles/en_US/FAQ/error-messages. [Accessed: 14-Dec-2017].

^4^Life with Kuri (2017). Available online at: https://www.heykuri.com/living-with-a-personal-robot (Accessed December 13, 2017)

^5^iRobot Home Robots (2018). Available online at: http://www.irobot.com/ (Accessed January 06, 2018).

^6^Neato Robotics. Available online at: https://www.neatorobotics.com/ (Accessed January 01, 2018).
